# Acute fungal endometritis in women with abnormal uterine bleeding: Clinical and microbiological insights

**DOI:** 10.22034/cmm.2025.345248.1650

**Published:** 2025-08-03

**Authors:** Soheila Abbaszadeh Godarzi, Fatemeh Zahra Ranjbar Golafshani, Firoozeh Kermani, Saeid Mahdavi Omran

**Affiliations:** 1 Obstetrics and Gynecology Department, Faculty of Medicine, Babol University of Medical Sciences, Babol, Iran; 2 Infertility and Reproductive Health Research Center, Health Research Institute, Rouhani Hospital, Babol University of Medical Sciences, Babol, Iran; 3 Parasitology and Medical Mycology Department, Faculty of Medicine, Babol University of Medical Sciences, Babol, Iran; 4 Infectious Diseases and Tropical Medicine Research Center, Health Research Institute, Babol University of Medical Sciences, Babol, Iran; 5 Student Research Committee, Babol University of Medical Sciences, Babol, Iran

**Keywords:** Abnormal uterine bleeding, *Candida*, Endometritis, Fungal endometritis

## Abstract

**Background and Purpose:**

Fungal endometritis, an uncommon yet severe condition affecting the uterine lining, typically manifests with abnormal uterine bleeding (AUB), pelvic discomfort, and vaginal discharge. This investigation aimed to present a pioneering study focused on fungal endometritis in women presenting with these clinical symptoms.

**Materials and Methods:**

A cohort of 43 female patients experiencing abnormal uterine bleeding was comprehensively evaluated at Babol University of Medical Sciences in Babol, Iran, between March 21, 2023, and March 19, 2024. Diagnostic procedures encompassed ultrasound imaging, a range of laboratory tests, hysteroscopy for direct visualization and tissue sampling, microscopic examination, fungal culture with subsequent colony count, and polymerase chain reaction for enhanced accuracy in fungal identification. Additionally, drug susceptibility patterns were assessed for all isolated fungal species.

**Results:**

Among the 43 patients, five (11.62%) received a definitive diagnosis of fungal endometritis. The identified species included two isolates of *Candida albicans*, two isolates of *Nakaseomyces glabratus* (previously known as *C. glabrata*), and one isolate of *Candida orthopsilosis*. A notable diagnostic observation was that all confirmed cases yielded negative results from vaginal discharge cultures, emphasizing the necessity of direct endometrial sampling. Antifungal susceptibility testing revealed varying minimum inhibitory concentrations among the isolates, though all responded effectively to the combined treatment of voriconazole and surgical debridement.

**Conclusion:**

This study highlighted the critical importance of prompt evaluation and precise diagnosis, including comprehensive antifungal susceptibility testing, in individuals presenting with acute endometritis and AUB. Such rigorous considerations are essential for guiding clinical management and mitigating the risk of adverse outcomes, particularly given the increasing antifungal resistance and the emergence of non-*albicans Candida* species as significant pathogens.

## Introduction

Endometritis, an inflammatory and infectious disorder of the endometrium, is primarily categorized into two forms, namely acute and chronic [ 1 ]. Acute endometritis is histopathologically characterized by the formation of microabscesses and the infiltration of neutrophils within the superficial epithelium, glandular lumina, and uterine cavity, frequently correlating with abnormal uterine bleeding (AUB) [ 1 , 2 ]. In contrast, chronic endometritis is characterized by edematous changes, increased stromal cell density, distinct maturation discrepancies between the epithelium and stroma, and the infiltration of endometrial stromal plasma cells. While bacterial infections are the most prevalent cause of endometritis, fungal etiologies, although rare, represent a potentially severe manifestation of the disease [ 3 ].

Under physiological conditions, the human endometrium is dynamically regulated by the infiltration of various immune cells, including macrophages, natural killer cells, and diverse subsets of T lymphocytes. Composition and density of these endometrial immune cells exhibit periodic changes throughout the menstrual cycle [ 4 ]. These precisely timed fluctuations in local leukocyte subpopulations are believed to facilitate the tissue remodeling necessary for maintaining endometrial receptivity [ 5 ]. Disruption of this delicate immune balance can predispose the endometrium to infection.

Acute fungal endometritis, caused by fungal proliferation within the uterine lining, can lead to AUB through multiple mechanisms, including inflammation, impaired blood coagulation, and endometrial tissue overgrowth. *Candida* species, particularly *C. albicans*, are the most frequently implicated fungi in such infections [ 6 , 7 ]. Despite its potential severity, fungal endometritis remains an under-recognized condition, largely due to significant diagnostic challenges. Its symptoms often mimic those of other uterine pathologies, such as endometriosis and myomas, leading to misdiagnosis or delayed intervention [ 8 , 9 ].

A notable deficiency in contemporary clinical practice is the reliance on clinical evidence and symptomatology for diagnosis, often devoid of definitive histological or microbiological confirmation [ 10 ]. Acute fungal endometritis may contribute to AUB through various mechanisms, including inflammation, impaired hemostasis, and the proliferation of endometrial tissue [ 8 ].

This study aimed to address this critical knowledge gap by offering a comprehensive characterization of fungal endometritis. Although the term "first global investigation" may suggest an extensive epidemiological survey, the primary contribution of this study lay in its meticulous clinical and microbiological characterization of acute fungal endometritis in women presenting with AUB, with a particular emphasis on the fungal etiology. This rigorous methodology, despite being conducted with a limited cohort, held global significance as it established a precedent and framework for future inquiries into this rare condition, thereby enhancing our understanding of a poorly delineated clinical entity. The impetus for this investigation stemmed from the pressing need for early and accurate diagnosis, accompanied by appropriate individualized treatment, to avert severe complications and improve patient quality of life. This research aspired to yield crucial insights that would inform the clinical management of this complex condition.

## Materials and Methods

### Ethics approval and consent to participate

Written informed consent was obtained from the patient before the commencement of this research. The study was conducted by Babol University of Medical Sciences in compliance with the ethical guidelines specified under code IR.MUBABOL.HRI.REC.1402.285.

### Patients

This study was undertaken covering a period from March 21, 2023, to March 19, 2024, corresponding to the Iranian calendar years 1402 to 1403.

A cohort of 43 patients presenting with abnormal vaginal bleeding was meticulously evaluated. The inclusion criteria specifically targeted patients whose initial diagnostic tests yielded normal results and whose symptoms did not respond to standard pharmacological treatments, thereby necessitating further investigation to exclude malignancy, cancer, and other uterine infections.

All enrolled patients underwent a comprehensive clinical assessment. This encompassed a detailed medical history that involved a thorough evaluation of their bleeding patterns, including the duration of each menstrual period, the volume of bleeding, occurrences of inter-menstrual spotting, associated pain, and any recent changes in bleeding patterns. The medical history also included chronic illnesses, previous surgeries, current medications, and familial histories of gynecological disorders. Additionally, lifestyle factors such as stress levels, physical activity, dietary habits, and contraceptive methods were considered, as these elements can significantly influence bleeding patterns.

A physical examination, including a pelvic examination, was conducted to assess the size, shape, and position of the uterus and ovaries, as well as to identify any masses or tenderness in the pelvic region. Diagnostic tests comprised a complete blood count to evaluate for anemia, hormonal assessments to measure levels of thyroid hormones, progesterone, and estrogen, and coagulation studies to detect any blood coagulation disorders. Ultrasound imaging was employed to evaluate the size and morphology of the uterus and ovaries and to identify the presence of fibroids, cysts, or other lesions. An endometrial biopsy was also performed to obtain a tissue sample for microscopic analysis, primarily aimed at identifying malignancies and other histological abnormalities.

### Sampling

For endometrial tissue sampling, the vagina was thoroughly cleansed with 20 ml of iodine solution. The cervix was then gently grasped with colored forceps and extracted. The surgeon measured the length of the uterus using a uterine catheter (hysterometer) and inserted a dilator of the same length into the uterus to prevent damage to surrounding tissues. Following the induction of anesthesia, an endometrial biopsy was performed using a curette suction catheter (Unimar, Inc., Wilton, Conn.) [ 11 ].

Two distinct samples were obtained to ensure a comprehensive evaluation. The first sample, collected from the superficial tissue of the endometrium, was placed in a sterile container containing 10 ml of normal saline for general histological analysis. The second sample, obtained from deeper tissue, was placed in a separate sterile container with 10 ml of normal saline and immediately dispatched for fungal analysis. This dual sampling approach was critical for distinguishing between superficial contamination and true deep-seated infection, thereby enhancing the diagnostic yield.

### Direct Examination and Culture

For direct microscopic examination, three slides were prepared from the endometrial samples and stained with Methylene blue, Giemsa, and Gram stains, followed by microscopic examination. For fungal culture, the collected samples were inoculated onto Sabouraud dextrose agar medium (Merck, Germany) supplemented with 50 mg/l chloramphenicol (Merck, Germany) to inhibit bacterial growth. The plates were incubated at both 37 °C and 25 °C for 24 h. Plates that showed no growth after 24 h were further incubated for one week to ensure the detection of slow-growing fungal species.

Following incubation, colony-forming units were enumerated on the Sabouraud dextrose agar plates. This quantitative assessment of fungal burden was crucial for distinguishing true infection from potential contamination, providing a more robust basis for diagnosis. *Candida* species identification was performed by plating isolates on CHROMagar (Merck, Germany), with color and morphology recorded according to the instructions of the manufacturer. Fungal isolates were subsequently preserved in sterile 1.5 ml microtubes containing sterile distilled water and 20% glycerol at -20 °C for future molecular procedures.

### Polymerase chain reaction(PCR)

To enhance the precision and accuracy of fungal identification beyond culture, direct tissue DNA extraction was conducted [ 12 ]. Amplification of the Internal Transcribed Spacer (ITS) region was performed using the universal fungal primers ITS1 (5'TCCGTAGGTGAACCTGCGG3') and ITS4 (5'TCCTCCGCTTATTGATATGC 3'). Each 25 μl PCR reaction volume comprised 12.5 μl of Master Mix, 1 μl of extracted DNA from each isolate, and 10.5 μl of DEPC-treated water.

The thermocycling protocol included an initial denaturation step at 94 °C for 5 min, followed by 35 cycles of secondary denaturation at 94 °C for 1 min, annealing at 56 °C for 1 min, and extension at 72 °C for 1 min. A final extension step at 72 °C for 7 min concluded the reaction. Amplified PCR products were analyzed by 1.5% agarose gel electrophoresis and stained with Safe Stain. The PCR products were then sequenced, and the FASTA file sequences were modified, aligned, and submitted to GenBank (https://www.ncbi.nlm.nih.gov/genbank/) to obtain accession numbers. Afterward, they were compared to other reference strains from different sequences.

### Antifungal susceptibility test

*In vitro* susceptibility patterns of the isolated yeast species to various antifungal agents were evaluated according to the guidelines outlined in the Clinical and Laboratory Standards Institute M27-S4 document [ 13 ]. Inoculum volumes were prepared by harvesting cells from 24-hour cultures. The cell densities were adjusted spectrophotometrically to achieve optical densities ranging from 75% to 77% transmission, resulting in final inoculum sizes of 2.5×10^3^ to 5×10^3^ CFU/ml.

Antifungal agents were diluted in RPMI-1640 medium, which was buffered to pH 7.0 with 0.165 M morpholinepropanesulfonic acid, supplemented with L-glutamine, and did not contain bicarbonate. This process yielded twofold concentrations of the agents, dispensed into 96-well microdilution trays. The final concentration range for fluconazole was 0.064–64 μg/ml, with similar ranges applied for itraconazole, clotrimazole, miconazole, voriconazole, and ketoconazole. Minimum inhibitory concentration (MIC) results for all agents were visually determined after incubation at 35 °C for 24-48 h. For amphotericin B, the MIC was defined as the lowest concentration of the drug that completely inhibited growth. For the other agents, the MIC was defined as the concentration that resulted in a significant decrease in growth by more than 50%. The strain *C. parapsilosis* (ATCC 22019) was utilized as a quality control throughout the testing.

## Results

### Demographics

In total, 5 out of the 43 patients evaluated in this study (11.62%) were definitively diagnosed with Candida species fungal endometritis following a comprehensive exclusion of other contributing factors. Age range of patients diagnosed with fungal endometritis extended from 28 to 64 years, with a mean age of approximately 42.8 years. All patients had a history of at least one normal vaginal delivery, while one patient, aged 46 years, had also previously undergone a cesarean delivery.

### Clinical features

Duration of AUB among the confirmed cases exhibited considerable variability, ranging from a minimum of 1 month to a maximum of 2 years. Common symptoms reported by the patients included vaginal bleeding, abdominal pain, fever, and the accumulation of uterine fluid. Some cases also presented with additional symptoms, such as weight loss and urinary retention.

Several participants had underlying systemic diseases, including iron deficiency anemia, hypertension, diabetes, hyperthyroidism, and food allergies. Notably, two cases did not report any specific underlying conditions. The findings indicated that women of reproductive age, particularly those with a history of natural childbirth, diabetes, and other underlying health issues, may be more susceptible to fungal endometritis (Table 1).

**Table 1:** Demographic information of five patients with fungal endometritis

Yeast Species	Patient history				
	Age	Number /Method of deliveries	Duration of vaginal bleeding	Sign and symptoms	Underlying disease
***N. glabratus isolate 1***	28	1/NVD	8 months	Vaginal bleeding, endometrial polyps, and free fluid in the uterine cavity	Iron deficiency anemia and hypertension
***N. glabratus isolate 2***	46	2/NVD	2 years	Abdominal pain and vaginal bleeding	Hepatitis B surface antigen positive
		1/S/C			
***C. albicans isolate 1***	36	1/NVD	1 month	Abdominal pain and vaginal bleeding	Diabetes, hyperthyroidism, multiple gallstones, and food allergies
***C. albicans isolate 2***	40	3/NVD	2 years	Abdominal pain, fever, bleeding, urinary retention, and endometrial polyps	-
***C. orthopsilosis***	64	3/NVD	4 months	Vaginal bleeding	-

NVD: Natural Vaginal Delivery S/C: Cesarean section

### Microbiological findings

**Figure 1:**
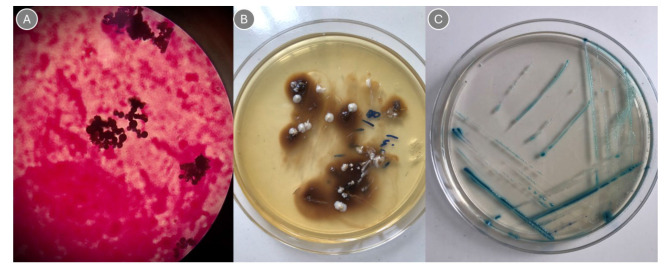
**A.** Budding yeast cell with gram staining at × 100 magnification **B.** Culture sample on Sabouraud Dextrose Agar medium **C.** *Candida albicans* culture sample on *Candida* CHROM Agar

In the five confirmed cases, Histopathological hematoxylin and eosin staining showed extensive inflammation. Based on the results of molecular tests, the fungal species identified included two isolates of *Candida albicans*, two isolates of *N. glabratus*, and one isolate of *C. orthopsilosis* (Figure 1).

A critical diagnostic observation was that all confirmed cases exhibited negative results in their vaginal discharge cultures. This finding strongly suggests the localized nature of the infection within the endometrium and underscores the inadequacy of superficial sampling methods for diagnosing fungal endometritis.

### Antifungal susceptibility test (AFST)

The MIC values for each isolate against various antifungal agents are presented in Table 2. Variations in MICs were observed among the different species and even among isolates of the same species. Despite these individual variations in MICs, all isolated species were ultimately susceptible to voriconazole, which was included in the effective treatment regimen administered to the patients.

**Table 2:** GenBank accession number(s) of five yeast species and *in vitro* MIC range antifungal susceptibility of six antifungal agents against five yeast species isolated from patients with fungal endometritis

Species	GenBank accession number	MIC (µg/ml)						
		FLU	MICO	CLO	KET	ITR	AMB	VOR
***N. glabratus* isolate 1**	PP930779	16	16	16	8	16	4	1
***N. glabratus* isolate 2**	PP930780	16	16	16	16	4	2	2
***C. albicans* isolate 1**	PP930776	16	16	16	4	4	8	0.25
***C. albicans* isolate 2**	PP930784	16	16	16	8	16	2	1
***C. orthopsilosis***	PP930778	16	8	16	8	8	1	0.5

FLU: fluconazole, MICO: miconazole, CLO: clotrimazole, KET: ketoconazole, ITR: itraconazole, AMB: amphotericin B, VOR: voriconazole, MIC: minimum inhibitory concentration

Following the diagnosis of the cause of the disease, all patients received antifungal treatment consisting of 200 mg of voriconazole oral tablet for a duration of 10 days. Subsequently, all patients were instructed to return to the medical center one month later for an evaluation of the progress of the treatment. This treatment regimen, in conjunction with curettage and debridement, effectively manages the disease. During the six-month follow-up, the physician evaluated the symptoms of patients, performed a pelvic examination, and ordered diagnostic tests, including ultrasound imaging and vaginal discharge cultures. Patients received instructions on hygiene practices and were advised to report any new or worsening symptoms. Effective management of fungal endometritis requires adherence to established hygiene protocols alongside medical treatment, which includes maintaining genital hygiene, avoiding gels, lotions, and scented cleansers, refraining from vaginal douching, and exercising caution with intrauterine devices. Incorporation of probiotics into the diet is also recommended.

## Discussion

Acute fungal endometritis in women with AUB, while infrequent, is typically not included in standard diagnostic evaluations [ 14 ]. This study was significantly important as it was characterized as the "first global study of fungal endometritis in symptomatic women," particularly those experiencing AUB. This pioneering aspect addressed a critical gap in the understanding of the global prevalence and clinical features of this condition. The successful treatment outcomes for patients in this study provide a compelling rationale for addressing this neglect. Failure to diagnose fungal endometritis may result in prolonged episodes of AUB, ineffective treatment regimens, and preventable complications [ 15 ]. The findings underscore the imperative to enhance clinical suspicion and incorporate specific diagnostic protocols for the accurate identification of this condition. This approach would facilitate prompt and effective treatment, ultimately improving patient outcomes that may otherwise be jeopardized due to misdiagnosis or delayed intervention [ 14 ].

Inner lining of the uterus, known as the endometrium, is inherently endowed with a sophisticated immune surveillance system. This protective barrier is sustained by a diverse array of immune cells, including macrophages, natural killer cells, and various subsets of T lymphocytes [ 16 ]. Population of endometrial immune cells is characterized by dynamic and periodic fluctuations in both composition and density throughout the menstrual cycle  [ 1 , 17 ]. These temporal changes are considered critical for facilitating tissue remodeling processes that are essential for achieving and maintaining endometrial receptivity, particularly for implantation [ 18 ]. In addition to cellular immunity, the uterine microecology is defined by a complex and dynamic interplay referred to as the “iron triangle,” which encompasses the endometrial microbiota, the local immune system, and the endometrial tissue itself. Within this intricate balance, commensal bacteria play a crucial role by competing with pathogenic organisms for ecological niches and by modulating the mucosal immune system, thereby enhancing the defense of the endometrium against infection [ 19 ].

Endometritis, characterized as an inflammatory and infectious disorder of the endometrium, arises when the protective equilibrium of this tissue is disrupted. Any alteration or deficiency in this precisely regulated endometrial immune system may result in compromised defense mechanisms, thereby predisposing the uterus to a range of pathological conditions, including infertility and adverse pregnancy outcomes. *Candida* species are widely acknowledged as opportunistic pathogens. Typically, they exist as symbionts on mucosal surfaces; however, they can transition to a pathogenic state when host immunity is compromised or when favorable environmental conditions emerge. Factors that predispose individuals to *Candida* infections include general immunosuppression, prolonged antibiotic use, and chronic metabolic conditions, such as diabetes mellitus, all of which are known to enhance *Candida* colonization in the vaginal environment [ 18 ].

The microorganisms identified in endometrial tissue frequently differ from those present in endocervical tissue or vaginal discharge. This observation suggests that microbial analyses performed on samples obtained from the lower genital tract may not serve as reliable predictors of the causative pathogens, as noted by Kotaro Kitaya *et al*. (2018) [ 20 ]. Reports have indicated that specific protozoa, helminths, and fungi possess the potential to disrupt female reproductive function [ 21 , 22 ]. These organisms possess the potential to induce abnormalities in the genital tract, thereby complicating conception and disrupting the normal processes of implantation and placental development [ 22 ]. Furthermore, fungal infections, specifically endometritis caused by *C. albicans*, are believed to be associated with infertility and AUB [ 20 ]. Additionally, some reports have suggested that *C. albicans* can have a negative impact on sperm motility [ 23 ].

The patient cohort in this study included individuals with various underlying conditions, such as iron deficiency anemia, hypertension, diabetes, hyperthyroidism, and food allergies. These factors, along with a history of vaginal or cesarean delivery, may significantly affect immune integrity or alter the uterine environment, making it more vulnerable to fungal overgrowth.

In this study, 11.62% of the patients were definitively diagnosed with fungal endometritis caused by Candida species, after other causative agents were carefully excluded. Identification of *C. albicans* alongside non-*albicans* species, such as *N. glabratus* and *C. orthopsilosis*, is a critical detail. While *C. albicans* has historically been the predominant Candida pathogen, available evidence suggests a “significant increase in non-*albicans* species” that have the potential for “inherent resistance to azoles,” making species identification critical for treatment [ 24 ]. This means that a diagnostic approach that focuses solely on *C. albicans* misses a significant proportion of cases or leads to inappropriate treatment of non-*albicans* infections [ 22 , 25 ]. Presence of N. glabratus, which is known for its resistance concerns, further reinforces this point. Therefore, the accurate species identification in this study challenges the traditional view of *C. albicans*-focused endometrial candidiasis [ 7 , 26 ].

This calls for a paradigm shift towards routine species-level identification in clinical microbiology to ensure appropriate and targeted antifungal therapy, thereby further reducing the risks of treatment failure and emergence of drug resistance. Laboratory assessments revealed a relatively similar drug susceptibility pattern among all species isolated, providing crucial insights for targeted therapy [ 27 ]. In clinical practice, AFST results are often unavailable or delayed, often forcing clinicians to initiate empirical antifungal therapy based on general guidelines or personal experience. However, given the increasing rates of antifungal resistance and the emergence of intrinsically resistant strains, it is of critical importance to perform AFST for all *Candida* strains causing invasive disease. This allows for targeted antifungal approaches and ensures selection of the most effective agent and optimal dose [ 28 ].

While the study observed a general “similar drug susceptibility pattern” among isolates, the precise MIC values revealed subtle but clinically significant differences between individual isolates of the same species. For example, the MIC for amphotericin B for *C. albicans* isolate 1 (2 μg/mL) differs significantly from that for isolate 2 (8 μg/mL). Similarly, *N. glabratus* isolates showed differences in susceptibility to ketoconazole and amphotericin B. These differences, particularly for potent drugs, such as amphotericin B, could directly impact the efficacy of treatment, especially in severe or resistant infections where a lower MIC may result in a better clinical response or less toxicity. According to Brook *et al*. (1982), amphotericin B has shown the highest efficacy as an antifungal drug for this particular infection [ 29 ]; however, more research in this area is needed.

The administration of oral voriconazole tablets, combined with curettage and surgical debridement, demonstrated clinical efficacy, and positive results were maintained at a six-month follow-up. As shown in a study performed by Ranjbar Golafshani *et al*. (2025), it is an effective treatment for this endometritis [ 14 ].

This underscores the critical importance of accurate and isolate-specific AFST. Such precise information is essential for optimizing patient-specific treatment regimens, particularly in the context of emerging resistance, and for ensuring the most effective utilization of available antifungal agents.

## Conclusion

The findings underscore the critical importance of prompt and precise diagnosis, including definitive identification of fungal species and comprehensive AFST. This is especially crucial given the challenges posed by antifungal resistance and the emergence of non-*albicans Candida* species as notable pathogens. Voriconazole treatment and surgical debridement highlight a promising therapeutic approach for managing acute fungal endometritis. 

This study has several limitations, particularly regarding the observed effectiveness of the treatment, which should be interpreted cautiously due to its preliminary nature and the small sample size. Further research with larger, multi-center cohorts is essential to validate these findings and establish optimal diagnostic and therapeutic guidelines for fungal endometritis. Nonetheless, this study represents an important first step, generating hypotheses and prompting clinicians to consider fungal etiology in cases of unexplained or refractory AUB. Such an approach will pave the way for more definitive research and ultimately improve patient care.
